# CSatDTA: Prediction of Drug–Target Binding Affinity Using Convolution Model with Self-Attention

**DOI:** 10.3390/ijms23158453

**Published:** 2022-07-30

**Authors:** Ashutosh Ghimire, Hilal Tayara, Zhenyu Xuan, Kil To Chong

**Affiliations:** 1 Department of Electronics and Information Engineering, Jeonbuk National University, Jeonju 54896, Korea; ashutosh@jbnu.ac.kr; 2 School of International Engineering and Science, Jeonbuk National University, Jeonju 54896, Korea; hilaltayara@jbnu.ac.kr; 3 Department of Biological Sciences, The University of Texas at Dallas, Richardson, TX 75080, USA; 4 Advanced Electronics and Information Research Center, Jeonbuk National University, Jeonju 54896, Korea

**Keywords:** drug–target interaction, binding affinity, attention, convolution neural network, deep learning, artificial intelligence, pharmacometrics, drug discovery and development, proteins, ligands

## Abstract

Drug discovery, which aids to identify potential novel treatments, entails a broad range of fields of science, including chemistry, pharmacology, and biology. In the early stages of drug development, predicting drug–target affinity is crucial. The proposed model, the prediction of drug–target affinity using a convolution model with self-attention (CSatDTA), applies convolution-based self-attention mechanisms to the molecular drug and target sequences to predict drug–target affinity (DTA) effectively, unlike previous convolution methods, which exhibit significant limitations related to this aspect. The convolutional neural network (CNN) only works on a particular region of information, excluding comprehensive details. Self-attention, on the other hand, is a relatively recent technique for capturing long-range interactions that has been used primarily in sequence modeling tasks. The results of comparative experiments show that CSatDTA surpasses previous sequence-based or other approaches and has outstanding retention abilities.

## 1. Introduction

Biochemically dysfunctional allosteric changes in proteins are frequently the cause of many diseases. A drug can change the way native proteins operate in the body, resulting in a desirable therapeutic effect [1]. However, the response of individual patients to a drug varies depending on genetic factors, and prescribers must recognize the need to monitor the outcomes of their prescription [2]. In addition, developing a new drug is expensive [3], and obtaining FDA clearance might require several years [4]. Because of the high cost of drug development, several pharmaceutical companies employ consumer research techniques similar to those used in other industries, in which the genuine value of a product is determined by its consumers [5]. The reduction in drug development costs will not only result in lower drug prices and healthcare expenses for patients, but also allow corporations to produce tailored drugs based on the genetics of individuals. Analyzing the vast extent of available drug and target data in existing databases, emerging and revolutionary computer technologies and deep learning concepts can lower drug development expenses. Currently, neural networks are considered to be relatively beneficial in bioinformatics applications [6,7,8,9,10].

The drug–target interaction identification is an essential aspect of the development of genetic drugs [11], as only the drugs and targets with similar molecular configurations are compatible [12]. The strength of the drug–target interaction pair was measured using DTA. DTA prediction is the essential phase in the computer-aided design (CAD) of a drug, which can accelerate drug development and limit the usage of resources. Drugs can affect medical conditions by attaching to proteins and can be beneficial or badly affect their functionality. The high binding affinity of a small molecule to a target protein is one factor in selecting a candidate component for drug development. Since the invention of deep learning, its application to DTA prediction to enhance accuracy has received considerable research attention [13]. The equilibrium dissociation constant ($K_{d}$) was utilized to evaluate and rank the strength of the interaction binding affinity. Besides $K_{d}$, binding affinity is expressed using the half maximal inhibitory concentration ($IC_{v}$) or the inhibition constant ($K_{i}$). The low ($K_{i}$) values are associated with the high binding affinity of the ligand to its target. Similarly, the weak attraction and binding between the target molecule and ligand result in a high $K_{d}$ value [14]. In addition, ($IC_{5}0$) values indicate high binding, which is dependent on the ligand and target concentrations [15].

Several studies have predicted DTA using various models for compounds and targets [16,17,18]. Deep learning models, most notably CNNs, which learn from protein–ligand composites’ three-dimensional (3D) organization, have also been used to score protein–ligand interactions. Some studies [19] have used the Smith–Waterman algorithm, which only employs similarity representations of the targets, and the KronRLS method two-dimensional (2D) compound similarity interpretation of the drugs. Because these techniques require the computation of the similarity matrix, they are constrained to known drug–target composite structures based on the 25,000 drugs in the lists of the Protein Data Bank (PDB) [20], thereby limiting the number of molecules used in the training process. Another study [21] developed a model, referred to as DeepDTA, using a one-dimensional (1D) representation of proteins and ligands to solve these constraints. Instead of using binding complexes’ 3D structures or the exterior characteristics, they applied protein sequences and compound representations using the Simplified Molecular Input Line Entry System (SMILES). Additionally, CNN blocks were used to detect bases from SMILES strings and raw protein sequences.

The convolutional neural network [22,23,24,25] is a widely used deep learning model that has already achieved significant progress in the realm of feature extraction. Despite possessing the potential benefits of a CNN-based technique, DeepDTA has severe limitations. Long-distance dependencies, for example, are difficult to capture with CNNs. Furthermore, given a raw molecular sequence, CNNs are unable to represent the possible interactions between distant atoms. The CNN model, for example, can record connections between atoms in a series of approximately 35 distances using three convolution layers, each with a filter size of 12. After extracting features from the convolution layers, we used the self-attention mechanism to capture the relationships between atoms in a sequence in our suggested model. Because a multi-head technique with each head corresponding to a feature subspace was used, the applied self-attention mechanism may attend to characteristic subspaces and spatial subspaces simultaneously, in contrast to previous approaches. The proposed model was tested on the popular standard datasets, Davis [26] and KIBA [27].

In contrast to prior deep learning models, our proposed technique results in the elucidation of a more accurate molecular connection. Furthermore, the attention mechanism was used to understand a molecule’s high-dimensional structure from a raw sequence. Our suggested technique surpasses existing approaches in terms of DTA prediction without depending on the complex’s 3D structure or a 2D representation of the molecule, according to the results. Our findings imply that the attention mechanism is more accurate in terms of abstract and meaningful concepts. Furthermore, we discuss the effective utilization of these discoveries for the further advancement of related research.

## 2. Result

We consider DeepDTA as the baseline method because it is a typical computational-nonstructure-based approach devised to predict DTAs using a CNN model. To compare our model to up-to-the-minute DeepDTA [21], KronRLS [28], SimBoost [29], WideDTA [30], and GraphDTA [31] models, we adopted the same KiBA [27] and Davis [26] dataset benchmarks. The Davis dataset’s binding intensities for 442 targets and 72 drugs were measured using Kd constants varying between 5.00 and 10.80. KiBA is a database that holds the binding interactions of 2116 drugs and 229 targets, which are represented by KiBA scores that vary between 0.00 and 17.20. To make the evaluation as unbiased as possible, we employed the same number of testing and training cases, as well as the same evaluation measures: CI, MSE, and RMSE. Small MSE and large CI values are ideal. The performance measures originally published in DeepDTA for all the baseline approaches are provided. Table 1 summarizes the hyperparameters used in the experiments. Multiple times, the hyperparameters were fine-tuned.

Our CSatDTA model predicts drug–target binding capacity using just drug and protein sequence information. The average MSE, RMSE, and CI values for the datasets KiBA and Davis are shown in Table 2 and Table 3, respectively. Previously, the best CI for the Davis dataset was obtained from the GAT-GCN model [31], which was 0.881. Similarly, the same model obtained a better MSE and RMSE, which were 0.245 and 0.494. The CI obtained from our model was 0.892, which is better than the previous performance on the Davis benchmark dataset. The MSE and RMSE were also better than the prior models, with values of 0.241 and 0.490, respectively. The best CI, MSE, and RMSE among the preceding models for the KiBA dataset were 0.891, 0.140, and 0.374, respectively, attained by the GAT-GCN model [31].

**Figure 1:**
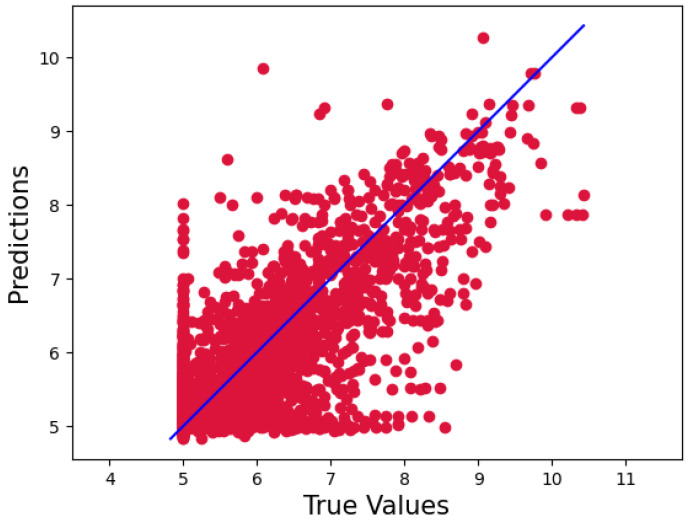
Predictions of the CSatDTA model vs. measured binding affinity values for the Davis dataset.

**Figure 2:**
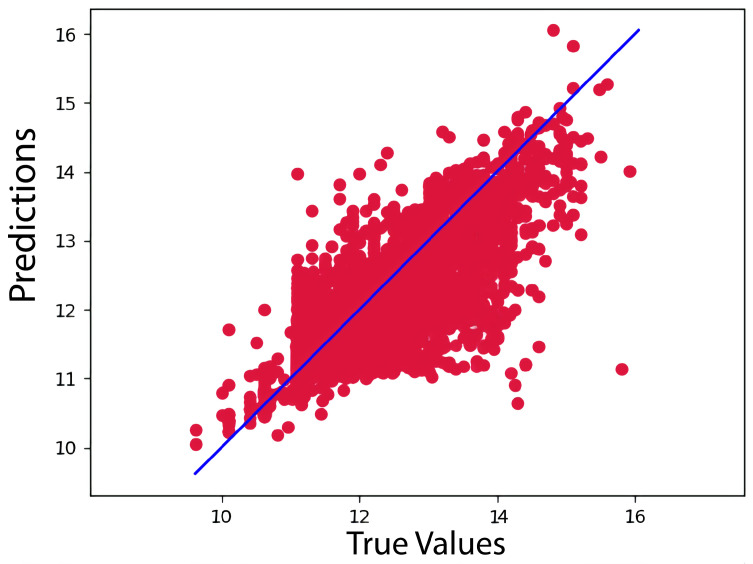
Predictions of the CSatDTA model vs. measured binding affinity values for KiBA.

Our model, however, outperformed the data for the KiBA dataset, with a CI, MSE, and RMSE of 0.898, 0.134, and 0.366, respectively. Because of its more comprehensive pharmacological library, the KiBA dataset outperformed the Davis datasets in this analysis. Moreover, this comes from making the strides as the dataset grows in size, since the KiBA dataset is four-times the size of the Davis dataset. The capacity of deep learning models to capture hidden knowledge improves as the amount of data increases. Figure 1 and Figure 2 depict scatter plots for the Davis and KiBA datasets, respectively.

Our findings show that attention enhancement leads to systematic gains in prediction and feature identification tasks across a wide range of architectures and computational methodologies. In addition, our experiments demonstrated the efficacy of the suggested 2D relative attention mechanism. We used self-attention feature maps instead of convolutional feature maps in all trials to allow straightforward comparisons with the baseline models. All outcomes were consistent with the 2D relative self-attention mechanism unless otherwise stated.

### Prediction Web Server

**Figure 3:**
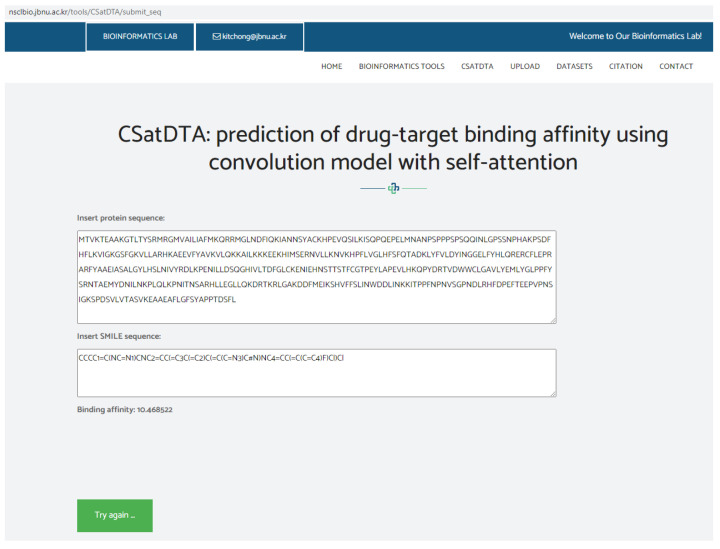
Snapshot of webserver showing binding affinity prediction.

A web server was used to implement the CSatDTA model. For the input, the tool accepts SMILES strings for the drugs and protein sequences for the targets to calculate the affinity score. Public access to the server is available at http://nsclbio.jbnu.ac.kr/tools/CSatDTA/ (accessed on 15 July 2022). Figure 3 depicts a snapshot of the server implementation showing the binding affinity prediction of SGK1 protein and a drug $C_{23}H_{19}Cl_{2}FN_{6}$.

## 3. Discussion

In this study, we used self-attention instead of convolutions for regression models. We presented a new 2D relative self-attention regression technique that allowed the training of completely competitive self-attention affinity prediction models using sequence data. We demonstrated that this self-attention mechanism outperforms alternative attention schemes and proposed it as an addition to convolutional operators. Furthermore, extensive tests showed that attention augmentation improves the previous convolution neural network approach in a systematic manner.

The results revealed that deep-learning-based approaches with an attention mechanism significantly outperformed baseline methods or the previous approaches with statistical significance. The research makes a significant contribution by presenting a unique deep-learning-based model that can predict drug–target compatibility that simply employs protein and drug character representations. For both drugs and targets, we achieved comparable or better results than baseline approaches, which depend on a variety of techniques and strategies to extract properties from raw sequence data. Our experiment predicted new interactions between known drugs and targets. In future studies, we will focus on predicting known targets for new drugs. In addition, we intend to extend this methodology to predict known drugs for novel targets.

## 4. Materials and Methods

### 4.1. Materials

To evaluate the proposed model, we used two datasets, KiBA and Davis, as standard data for comparison with the previous models. The disassociation constant ($pk_{d}$) values for approximately 120,000 interactions are included in the KiBA dataset, with 69 percent of them having affinity values of 10,000 nM ($pk_{d}$ = 5), suggesting weak or no interactions. The Davis dataset is smaller than KiBA, with only about 30,000 interactions.The KiBA values are derived from various data sources, including $IC_{5}0$, $K_{i}$, and $K_{d}$, whereas Davis is derived exclusively from $K_{d}$. The number of drugs, targets, and interactions, as well as the statistics of the datasets are provided in Table 4.

### 4.2. Drug and Target Representation

**Figure 4:**
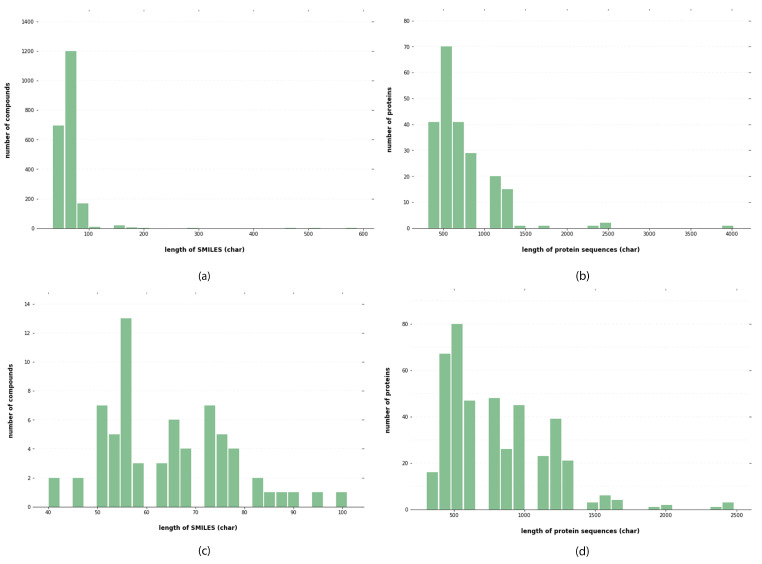
Analysis of KiBA and Davis datasets: (**a**) distribution of length of SMILES for KiBA datasets, (**b**) distribution of length of protein sequences for KiBA datasets, (**c**) distribution of length of SMILES for Davis datasets, and (**d**) distribution of length of protein sequences for Davis datasets.

For drugs, the input is fed as SMILES data, a form readable by computers [32]. The SMILES are parsable with a context-free parser. This representation has been used while predicting biochemical properties, including toxicity and biodegradability, based on the fundamental principle of cheminformatics, that similar molecules have similar properties [11]. With the SMILES code, drug properties such as the bulky atom number or amount of valence electrons can be retrieved and used as attraction prediction features. A string representation of the SMILES code can be found here. Natural language processing (NLP) techniques or a deep learning model can be used to highlight the strings. SMILES data for all compounds were collected from the PubChem database. After obtaining the SMILES string, it was passed through a unique label representation of the strings. In total, 64 labels were utilized. Each label is represented with corresponding integers. The data distribution from the Davis and KiBA datasets is shown in Figure 4.

Proteins are represented using one-hot encoding. For each target in the experimental datasets, the UniProt database was used to retrieve protein sequences with the help of the protein gene name. A series of ASCII characteristics representing amino acids constituted the sequence. Each amino acid type was assigned an integer based on its alphabetical symbol, and proteins are represented as an integer sequence. Aspartic acid (D) is four; alanine (A) is one; cysteine (C) is three. The sequence was shortened or padded to a preset length of 1000 residues to make training easier. When a sequence was too short, zero values were inserted to extend it. Similarly, we determined a maximum character length of 100 for SMILES. The maximum length was determined by the distribution lengths of protein sequences and SMILES, as shown in Figure 4, to encompass at least 90% of the protein sequence and 80% of the SMILES compounds. Sequences shorter than the maximum limit were padded with zeros, whereas longer ones were terminated.

### 4.3. Proposed Prediction Model: CSatDTA

This research proposes CSatDTA, a self-attention augmented CNN-based model. The proposed model is motivated by the fact that target sequences and the structure of drugs are very comparable to natural language texts, in which atomic information, both structural and contextual, is crucial for comprehending the attributes of a molecule. The molecular structure of substances is encoded by SMILE sequences in which each atom interacts with both distant and nearby atoms. However, when representing a molecule, the current DeepDTA approach [21] employing CNNs is unable to link long-distance atoms. We used the self-attention technique to overcome this issue. Some studies have shown that combining convolutions with self-attention produces the optimum results [33]. In computer experiments, we found that the self-attention-aided convolution method outperforms convolutions as a stand-alone computational primitive in the DTA. We describe the CSatDTA model to enhance convolutional operators using the self-attention mechanism by concatenating feature maps of convolutions with a collection of feature maps created by self-attention. First, we discuss the input and output representations for the proposed CSatDTA model architecture. Following an explanation of the model training procedure, we go through the basic building blocks of the CSatDTA model.

**Figure 5:**
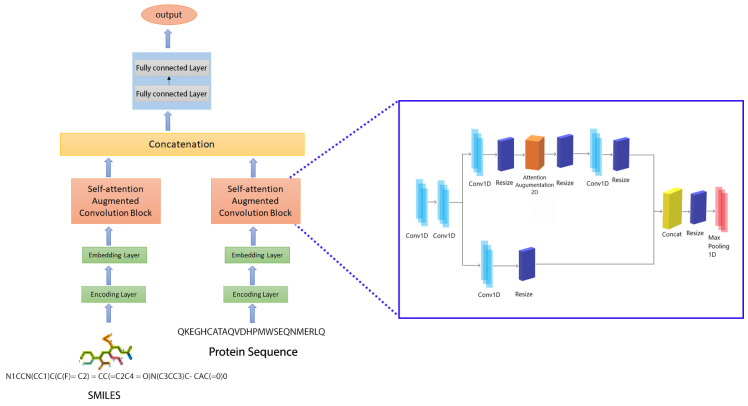
The proposed model’s architecture.

As a regression problem, we computed the binding strength score from drug and target interaction forecasting in this work. As for estimation analytics, we chose a well-known deep neural network: a CNN with a self-attention technique, as shown in Figure 5. A CNN is a sort of architecture that consists of one or more convolutional layers, as well as a pooling layer. A max-pooling downsamples the previous layer’s findings, allowing for the generalization of the characteristics acquired by the filters. The performance of the model was enhanced by an attention mechanism in addition to the CNN. A weighted average of the values computed using the concealed units was produced by self-attention. Our attention-augmented networks use self-attention throughout the design rather than pre-training, similar to their fully convolutional competitors. The multi-head attention (MHA) mechanism allows the model to give importance to both the spatial and feature subspaces at the same time. Furthermore, we extended the relative self-attention to 2D inputs, enabling us to model equivariance systematically, improving the representational capacity of self-attention. Instead of adding or reducing convolutional features, our method generates additional feature maps. This ability enables us to dynamically modify the proportion of attentional channels and evaluate a range of designs, from fully convolutional to attentional models.

Fully connected (FC) dense layers were added to the model in addition to the convolutional, attention, and max-pooling layers. The potential of convolutional networks to extract local dependencies using filters is their most significant feature. As a consequence, the size and number of filters in a CNN have a direct impact on the type of characteristics the model extracts from the feed. It is commonly assumed that as the number of filters in a model grows, so does the model’s capacity to recognize patterns. We suggested a prediction model based on the attention CNN that consists of two self-attention-augmented convolutional blocks. In this study, $d_{k}$, $d_{v}$, and $N_{h}$ relate to the depth of keys, the depth of values, and the number of heads in MHA, respectively. We also assume that $d_{k}$ and $d_{v}$ can be uniformly divided by $N_{h}$ and denote $d_{k}^{h}$ and $d_{v}^{h}$, respectively, as the depth of keys and values per attention head. Each convolutional block with self-attention augmentation seeks to learn representations from protein sequences/SMILES strings. We used an architecture of five convolution layers and one attention layer for each block.

The number of filters in the first two 1D convolutional layers increases with the number of layers, with the second layer having twice as many as the first. However, for the remaining convolution layers, the number of filters was manipulated with $d_{k}$ and $d_{v}$ in such a way that the final layer was reshaped to three-times the filter number in the first layer. A max-pooling layer then followed the neural network blocks. The max-pooling layers’ final features were concatenated and then fed into the FC layers. In the first and second dense layers, we employed 1024 nodes each, which was accompanied by a 0.1 dropout layer. Dropout is a regularization strategy that involves turning off the activation of particular neurons to minimize overfitting [34]. The output layer was followed by the third layer, which had 512 nodes. Figure 5 depicts the proposed model, which combines two CNN blocks.

#### 4.3.1. Attention Mechanism

We used the Transformer architecture [35] to flatten an input tensor of shape (H,W,Fin) to a matrix $x\in R^{HW\times F_{in}}$ and conduct the MHA. For a single head h, the output of the self-attention mechanism can be described as:

$$O_{h}=\mathrm{Softmax}\left(\frac{\left(XW_{q}\right){\left(XW_{k}\right)}^{T}}{\sqrt{d_{k}^{h}}}\right)\left(XW_{v}\right).$$

where $W_{v}\in R^{F},W_{q}$ and $W_{k}\in R^{F_{in}\times d_{k}^{h}}$ are learned linear maps that convert the input *X* to queries Q = $XW_{q}$, values V = $XW_{v}$, and keys K = $XW_{k}$. After that, the outputs of all heads are combined and provided again as follows:

$$\mathrm{MHA}\left(X\right)=\mathrm{Concat}\left[O_{1},\ldots ,O_{Nh}\right]W^{o}$$

where $W^{o}\in R^{d_{v}\times d_{v}}$ is a linear map that has been learned. To equal the pioneer dimensions, MHA(X) is reshaped into a tensor of shape $\left(H,W,d_{v}\right)$.

#### 4.3.2. Combining Attention and Convolutional Feature Mapping

Consider an original convolution operator with an $F_{out}$ output filter, $F_{in}$ input filters, and filter size *k* in formal terms. This corresponds to attention-enhanced convolution, which can be written as:

AAConv(X)=Concat[Conv(X),MHA(X)]

The ratio of key depth to the number of pioneer output filters is denoted by $k=\frac{d_{k}}{F_{out}}$, while the ratio of attentional channels to the number of pioneer output filters is denoted by $v=\frac{d_{v}}{F_{out}}$. The suggested attention-enhanced convolution is translation equivariant, like convolution, and may easily operate on inputs of many spatial dimensions.

#### 4.3.3. Impact on the Number of Parameters

The MHA uses a linear convolution layer $\left(2d_{k}+d_{v}\right)=F_{out}\left(2k+v\right)$ output and $F_{in}$ input kernels, as well as an added linear convolution layer with $d_{v}$ = $F_{out}$ input and output kernels to mix the contributions of different heads for the computation of keys, values, and queries. After the number of filters in the convolutional section is reduced, the following changes occur in the parameters:

$$\Delta_{params}∼F_{in}F_{out}\left(2k+\left(1-k^{2}\right)v+\frac{F_{out}}{F_{in}}v^{2}\right)$$

For simplicity, the arguments introduced by relative position embeddings are ignored, benefiting from their negligibility. In practice, this results in a minor decrease in parameters when 3 × 3 convolutions are replaced and a slight increase in parameters for the replacement of 1 × 1 convolutions. Attention-augmented networks surpass fully convolutional networks by employing fewer test parameters, which is significant.

#### 4.3.4. Attention Augmented Convolutional Architectures

In this analysis, we used boosted convolution with a batch normalization layer [36], which can adapt to adjust the convolution and attention feature mapping contributions. We utilized our boosted convolution per each residual block, similar to existing visual attention approaches [37,38]. In addition, we employed a limited batch size and often downsampled the inputs given to the self-attention layer. This lowers the memory consumption of attention-enhanced networks.

### 4.4. Evaluation Metrics

For evaluating the performance of these models, we applied the following metrics.

#### 4.4.1. Concordance Index

The concordance index (CI) is considered as an analysis statistic to assure DTA predictive performance, as mentioned earlier [4]. The CI [17] is a ranking indicator for continuous data. The CI was used to measure how well the binding strength values of protein–ligand interactions are predicted with respect to the real values. The CI extends from $\frac{1}{2}$ to 1, with 1 denoting perfect prediction accuracy and $\frac{1}{2}$ denoting a random predictor. The following formula was used to calculate the CI:

$$\mathrm{CI}=\frac{1}{Z}\sum_{\delta_{i}>\delta_{j}}h\left(b_{i}-b_{j}\right)$$

$s_{i}>s_{j}$, where $b_{i}$ is the greater affinity’s ($\delta_{i}$) predicted value, whereas for the smaller affinity ($\delta_{j}$), $b_{j}$ is the prediction value. Here, (*Z*) is a normalization constant that equals the number of data pairings with different label values. The Heaviside step function is represented by *h*(*x*), which is a discontinuous function defined as

$$h\left(x\right)=\left\{\begin{array}{cc} 1.0, & x>0\\ \frac{1}{2}, & x=0\\ 0.0, & x<0 \end{array}\right.$$

#### 4.4.2. Mean-Squared Error

For continuous prediction errors, the MSE is a commonly used statistic parameter. The MSE, such as the variance, is expressed in the same units as the estimated quantity’s square. The root-mean-squared error (RMSE or RMSD) is obtained by taking the square root of the MSE, which has the same units as the predicted quantity. The RMSE equals the square root of the variance, often known as the standard error, for an unbiased estimator. Because this is a regression task, we picked the MSE as the statistic.

$$\mathrm{MSE}=\frac{1}{n}\sum_{i=1}n{\left(p_{i}-y_{i}\right)}^{2}$$

where $p_{i}$ refers to the prediction, *n* to the number of samples, and $y_{i}$ to the actual output

#### 4.4.3. Root-Mean-Squared Error

The RMSE, which is among the linear regression measurements used in this study, is the mean distance between the predicted line and actual data. It is calculated as the square root of the MSE:

$$\mathrm{RMSE}=\sqrt{\mathrm{MSE}}$$

## Figures and Tables

**Table 1:** Hyperparameters for CSatDTA.

Hyperparameters	
Learning rate (initially)	0.001
Batch size	64
Optimizer	Adadelta
Kernel initializer	Glorot Normal
CNN layers	2
Attention layers	2
Number of attention heads for SMILES	4
Number of attention heads for proteins	10
Filters for keys and values for SMILES	2
Filters for keys and values for proteins	5

**Table 2:** Performance on the KiBA dataset in terms of prediction.

Method	Compound Rep.	Protein Rep.	MSE	RMSE	CI
KronRLS	Pubchem-Sim	Smith–Waterman	0.411	0.641	0.782
SimBoost	Pubchem-Sim	Smith–Waterman	0.222	0.471	0.836
DeepDTA	1D	1D	0.179	0.423	0.863
WideDTA	1D + LMCS	1D + PDM	0.194	0.440	0.875
GAT_GCN	Graph	1D	0.140	0.374	0.891
CsatDTA (Proposed)	1D	1D	0.134	0.366	0.898

**Table 3:** Performance on the Davis dataset in terms of prediction.

Method	Compound Rep.	Protein Rep.	MSE	RMSE	CI
KronRLS	Pubchem-Sim	Smith–Waterman	0.379	0.615	0.871
SimBoost	Pubchem-Sim	Smith–Waterman	0.282	0.531	0.872
DeepDTA	1D	1D	0.261	0.510	0.878
WideDTA	1D + LMCS	1D + PDM	0.262	0.511	0.886
GAT_GCN	Graph	1D	0.245	0.494	0.881
CsatDTA (Proposed)	1D	1D	0.241	0.490	0.892

**Table 4:** Datasets.

	Proteins	Compounds	Interactions
KIBA	229	2111	118,254
Davis	442	68	30,056

## Data Availability

The dataset used in this work can be downloaded from the project web page at http://nsclbio.jbnu.ac.kr/tools/CSatDTA/ (accessed on 15 July 2022).

## Abbreviations

The following abbreviations are used in this manuscript:

CSatDTA	Prediction of drug–target affinity using convolution model with self-attention
DTA	Drug–target affinity
CNN	Convolutional neural network
PDB	Protein Data Bank
MSE	Mean-squared error
CI	Concordance index
FC	Fully connected
MHA	Multi-head attention
SMILES	Simplified Molecular Input Line Entry System
